# Clinical evaluation of a plasma Neurofilament Light chain electrochemiluminescence immunoassay in Alzheimer’s disease

**DOI:** 10.1002/alz.091850

**Published:** 2025-01-09

**Authors:** Hadia A Fatima, Nikhil Padmanabhan, Rachel Cohen, Daniel Romero, Catherine Demos, Jacob N Wohlstadter, George Sigal, Steven E. Arnold, Pia Kivisäkk

**Affiliations:** ^1^ Massachusetts General Hospital, Boston, MA USA; ^2^ Meso Scale Diagnostics, LLC., Rockville, MD USA; ^3^ Massachusetts General Hospital, Harvard Medical School, Boston, MA USA; ^4^ Athinoula A. Martinos Center for Biomedical Imaging, Massachusetts General Hospital, Harvard Medical School, Charlestown, MA USA; ^5^ Massachusetts Alzheimer's Disease Research Center, Charlestown, MA USA

## Abstract

**Background:**

Neurofilament light chain (NfL) is a key biomarker for axonal injury providing diagnostic and prognostic value in many neurodegenerative diseases. The sensitivity of electrochemiluminescence (ECL) immunoassays has previously not been adequate to quantify plasma NfL levels in Alzheimer’s disease (AD). Here we performed clinical evaluation of a recently developed ultra‐sensitive plasma NfL ECL S‐PLEX^®^ immunoassay in individuals with AD and other neurodegenerative diseases.

**Method:**

We measured NfL in banked plasma samples from 541 participants in the Massachusetts ADRC’s longitudinal cohort. Of these, 262 had a diagnosis of AD, 121 non‐AD neurodegenerative diseases (Ndgen), 46 other neurological diseases (OND), and 112 were controls (CON). The clinical diagnoses (AD/no AD) were verified by AD biomarkers (neuropathology, amyloid PET, CSF amyloid‐β 42/40 ratio, and/or plasma pTau181). Longitudinal analysis identified 223 participants with mild cognitive impairment (CDR 0.5) at baseline and 4 years of follow up. Of these, 131 remained stable with unchanged CDR, and 92 declined with worsening cognitive impairment (CDR³1.0). NfL was measured in all participants using an ECL immunoassays from Meso Scale Discovery (MSD) and in 72 participants using a SIMOA assay from Quanterix.

**Result:**

NfL levels measured using the two assays were highly correlated. Cross‐sectional analysis using the MSD assay showed the expected differences between the diagnostic groups with highest NfL levels in the Ndgen group, followed by AD, and the two control groups. NfL levels correlated with disease severity measured by cognitive testing (MoCA, global CDR, CDR SOB, and FAQ) at the time of blood collection. NfL levels at baseline were furthermore higher in participants who declined during follow‐up compared to stable participants both when studying the whole cohort and when limiting the analysis to participants with a final diagnosis of AD.

**Conclusion:**

The ultrasensitive ECL plasma NfL assay demonstrated utility for detection of AD and correlated with disease severity. Moreover, baseline plasma levels of NfL reflect cognitive decline over the next 4 years, providing prognostic information that may have utility in both clinical practice and clinical trial populations.

